# A systematic review of the feasibility, acceptability, and efficacy of online supportive care interventions targeting men with a history of prostate cancer

**DOI:** 10.1007/s11764-018-0729-1

**Published:** 2019-01-04

**Authors:** Cynthia C. Forbes, Amy Finlay, Megan McIntosh, Shihab Siddiquee, Camille E. Short

**Affiliations:** 1 Hull York Medical School, University of Hull, Allam Medical Building 3rd Floor, Cottingham Road, Kingston-Upon-Hull, East Yorkshire HU6 7RX UK; 2 School of Health and Human Performance, Dalhousie University, Halifax, Nova Scotia Canada; 3 School of Medicine, Freemasons Foundation Centre for Men’s Health, University of Adelaide, Adelaide, Australia; 4 College of Medicine and Public Health, Flinders University, Adelaide, Australia; 5 School of Medicine, Freemasons Foundation Centre for Men’s Health, University of Adelaide, Adelaide, Australia

**Keywords:** Digital health, Cancer care, Men’s health, Well-being, Cancer services

## Abstract

**Purpose:**

To examine the feasibility, acceptability, and efficacy of online supportive care interventions targeting prostate cancer survivors (PCS).

**Methods:**

Studies were identified through structured searches of PubMed, Embase and PsycINFO databases, and bibliographic review. Inclusion criteria were (1) examined feasibility, acceptability, or efficacy of an online intervention designed to improve supportive care outcomes for PCS; (2) presented outcome data collected from PCS separately (if mixed cancer); and (3) evaluated efficacy outcomes using randomized controlled trial (RCT) design.

**Results:**

Sixteen studies met inclusion criteria; ten were classified as RCTs. Overall, 2446 men (average age 64 years) were included. Studies reported on the following outcomes: feasibility and acceptability of an online intervention (e.g., patient support, online medical record/follow-ups, or decision aids); reducing decisional conflict/distress; improving cancer-related distress and health-related quality of life; and satisfaction with cancer care.

**Conclusion:**

We found good preliminary evidence for online supportive care among PCS, but little high level evidence. Generally, the samples were small and unrepresentative. Further, inadequate acceptability measures made it difficult to determine actual PCS acceptability and satisfaction, and lack of control groups precluded strong conclusions regarding efficacy. Translation also appears minimal; few interventions are still publicly available. Larger trials with appropriate control groups and greater emphasis on translation of effective interventions is recommended.

**Implications for Cancer Survivors:**

Prostate cancer survivors have a variety of unmet supportive care needs. Using online delivery to improve the reach of high-quality supportive care programs could have a positive impact on health-related quality of life among PCS.

## Introduction

Prostate cancer is the most prevalent cancer (excluding nonmelanoma skins cancers) among men in many developed countries around the world [1]. Advances in screening and treatment technology in the past 30–40 years have significantly improved the 5-year survival rate of prostate cancer from around 68% to present rates of over 90% [2]. Though survival rates are high, quality of life (QoL) during survivorship may be poor. Prostate cancer treatment has been associated with numerous physical and psychological short- and long-term side-effects that have a significant impact on QoL [3, 4]. For those men diagnosed with or having developed advanced prostate cancer (with a 5-year survival rate of 29% [5]), the effects of treatments and dealing with a poor prognosis have even larger affects [6].

In fact, a large proportion of men with prostate cancer have reported functional and psychosocial supportive care needs; many of which are going unmet [7–9]. Supportive care needs can be defined as the requirements for care during and after treatments to help manage potential symptoms and side effects, help adaptation and coping, facilitate understanding and inform decision-making, and reduce or minimize functional declines [10]. A recent review examined the supportive care needs of prostate cancer survivors and found some of the most commonly reported are related to intimacy, information, physical, and psychosocial needs [9]. A review of supportive care interventions for men with prostate cancer concluded that interventions with combinations of educational, cognitive-behavioral, communication, and peer support were generally effective among intervention completers [11]. However, only 40% of interventions indicated acceptable mean attendance, and one-quarter of intervention effects were moderated by sociodemographic or psychosocial variables [11]. From a public health point of view, this suggests the need to improve intervention reach and adherence, while also ensuring interventions are sufficiently tailored to address unique needs, which are influenced by individuals’ sociodemographic and psychosocial profiles.

It has been suggested that utilizing online delivery of supportive care interventions may help to improve reach, while also allowing for high quality tailored care at a low cost [12–15]. Firstly, online delivery allows anonymity. This means men are able to share their feelings or experience without fear of being emasculated [16–18]. It also allows men to benefit from observing discussions of others if they are not interested in active participation [19, 20]. Secondly, online delivery can provide increased access to a multidisciplinary team of health professionals, without the need to leave home. This may be especially important for those living in rural or remote areas, where access to urban treatment centers can be difficult. Online delivery also affords the convenience of participation at any time of day allowing patients to access care outside of regular office hours [21, 22]. Finally, compared with other distance-based approaches (e.g., print-materials, DVDs, telephone calls), online approaches have the capacity to provide not only high-quality content, but also highly tailored content. This, in addition to opportunities for interaction with others and tools designed to support self-management and decision-making [23] favors online delivery.

Recent reviews have begun to examine the utility of technology and online delivery in follow-up and supportive care interventions for cancer survivors [12, 13, 15]. While results have been promising, the utility of online interventions for supporting men with prostate cancer remains somewhat unclear. To date, reviews have focused on mixed cancer samples only, and have not explored cancer type as a moderator or reported prostate cancer specific results. This limits conclusion regarding the acceptability, feasibility, and efficacy of online supportive care interventions among men with prostate cancer, especially given that breast cancer survivors are typically over represented. For example, recent reviews examined the types and efficacy of online interventions that reported QoL or QoL-related health outcomes and the effect of eHealth in physical activity promotion among various cancer survivor groups [15, 24]. McAlpine and colleagues advocate for multidimensional interventions that incorporate methods for educating participants and allowing participants to interact with each other and health care providers [15]. However, of the 14 studies they identified, only two reported results for prostate cancer survivors [25, 26] and the review omitted any descriptive feasibility and user acceptance (satisfaction) data that was included in the studies. To inform the development and or dissemination of online supportive care interventions targeting men with prostate cancer a detailed synthesis of the prostate cancer specific research is warranted.

This review aims to examine the feasibility, acceptability, and efficacy of online supportive care interventions targeting prostate cancer survivors. For the purpose of this review, online supportive care interventions are defined as interventions delivered via the internet (e.g., using a website, tablet, or mobile app) with the aim of meeting the informational, emotional, spiritual, social, and/or physical needs of patients during their diagnostic, treatment, or follow-up phase [27]. Patients are considered prostate cancer survivors if they have ever received a prostate cancer diagnosis. This includes those who are on active surveillance, those treated with curative intent and living disease free and those living with advanced prostate cancer. As the main aim of this review is to examine the potential of online interventions for delivering supportive care in any form, we have purposefully not targeted a specific stage of disease or treatment.

## Method

The protocol for this review was registered a priori with PROSPERO (ID CRD42017056319). The conduct and reporting of the review adheres to the Preferred Reporting Items for Systematic Reviews and Meta-analyses guidelines [28]. A standardized form (based on ERC Cochrane template for intervention reviews [29]) was used to extract and review all data. A copy of the form is available via our open science framework page (http://osf.io/unj5m).

### Eligibility criteria

Inclusion and exclusion criteria were established a priori before conducting database searches. Studies were eligible for inclusion if they (1) examined the feasibility, acceptability, or efficacy of at least one online intervention designed to improve supportive care outcomes for prostate cancer survivors as a major part of the study; (2) presented outcome data collected from prostate cancer survivors only; and (3) evaluated efficacy outcomes using a randomized trial and/or feasibility/acceptability using a single-arm or randomized trial design. Studies were excluded if (1) they included mixed samples (e.g., survivors and caregivers, or survivors of mixed cancer types) and did not report study outcomes specifically for prostate cancer survivors; (2) the intervention being evaluated was targeted primarily at clinicians or caregivers rather than prostate cancer survivors; (3) findings were only explored using qualitative research methods; (4) findings were published in any language other than English; or (5) if findings were available as a conference abstract only.

### Search strategy

Studies were primarily identified through a structured search of all publication years (until April 6, 2017) in the following electronic databases: PubMed, Embase, and PsycINFO. The search strategy was developed in consultation with a specialist librarian at the University of Adelaide. Mesh terms in PubMed and equivalent terms in other databases were identified and used to search for all key concepts. Searches restricted to abstract and title were also undertaken for selected keywords. Boolean logic was used to combine the terms. The search strategy was piloted and refined in each database to achieve a balance between sensitivity (identifying high numbers of relevant articles) and specificity (identifying a low number of irrelevant articles) [30]. As a result, in PubMed and Embase, search terms relating to prostate cancer AND ehealth AND intervention evaluation AND supportive care outcomes were searched. Whereas, in PsycINFO, only terms related to prostate cancer AND ehealth AND intervention evaluation were searched. The search terms used for each database are detailed in additional file 1. The database searches were conducted by a single author (CES). In addition to the database search, endnote libraries of authors were reviewed and citation chaining was employed to identify additional articles of interest [30].

### Study selection

All articles identified through the databases and hand searches were imputed into a citation manager. Duplicate records were then counted and removed. Two authors (CES and CF) independently screened all articles against the inclusion and exclusion criteria using a standardized form [29], taking title, abstract, and full-text into account. Any disagreements were discussed and resolved by consensus.

### Data extraction

A data extraction form was developed by the research team to extract information about the study setting, participant characteristics, study design, intervention characteristics, data collection methods, and findings relating to feasibility, acceptability, and efficacy of the intervention. Feasibility and acceptability data was extracted for all included studies were reported. Efficacy data was only extracted for randomized trials. In cases where pilot data and definitive RCT data were both available (and focused on the same outcomes) only RCT data was extracted. If findings were unclear based on results reported in the manuscript, corresponding authors were emailed and asked to provide clarification.

The extraction form followed a recommended template [29] and was pilot tested by two reviewers (CF, CES) independently (on three included articles) to ensure it captured all relevant information and was easy to use. Minor changes were made after reviewing the first two articles, and no further changes were considered necessary after reviewing the third article. Data were then extracted using the form by a single reviewer (CF or CES). A second reviewer randomly selected four articles (i.e., 25%) and reviewed the data extracted (CF or CES). As there were no discrepancies, data extraction by a second reviewer for the remaining articles was considered unnecessary.

### Methodological review

Methodological quality was assessed independently by two reviewers (CES and MM or CF and AF) using an existing tool [31]. Minor modifications to the tool were made to reflect current best practice recommendations regarding confounders in randomized trials [32, 33] and practical considerations surrounding blinding in psychological and health service research. Specifically, the risk of bias for confounding was based on whether likely confounders were accounted for at randomization or during data analysis, regardless of differences in participant characteristics at baseline. As blinding is difficult in this area, studies were given a ‘moderate’ rating by default [34]. Additionally, bias relating to dropout was assessed based on the immediate post-intervention follow-up rather than the final data collection point. This was to ensure that studies containing multiple follow-up points were not systematically rated as more biased compared to studies only reporting immediate post-intervention outcomes. Bias relating to data collection methods was assessed based on the primary outcome of interest for randomized controlled trials and for the main acceptability outcome measure for all other designs. All discrepancies were resolved by consensus.

### Outcomes

The following study integrity and recruitment feasibility outcomes were assessed: (1) the number of participants to enter the study, (2) reported recruitment obstacles, (3) representative samples, (4) if the intervention was implementation as intended, and (5) cost of implementation. Acceptability outcomes assessed included (1) intervention adherence rates; (2) assessments of participant engagement, acceptability, and appeal; (3) any intervention burden; and (4) number of adverse events. As with previous research, a 40% recruitment rate, 70% retention rate, and a 70% average attendance rate were deemed acceptable cut-points to assess feasibility [11, 35]. Outcomes relating to efficacy were varied and depended on the focus of the intervention. In each case, the change in supportive care outcome relative to the comparison group was reported. Efficacy outcomes were reported for randomized controlled trials (RCTs) only. Current availability of online interventions was determined by visiting the study URL, if included in the study, or by web search.

## Results

### Study selection

**Fig. 1:**
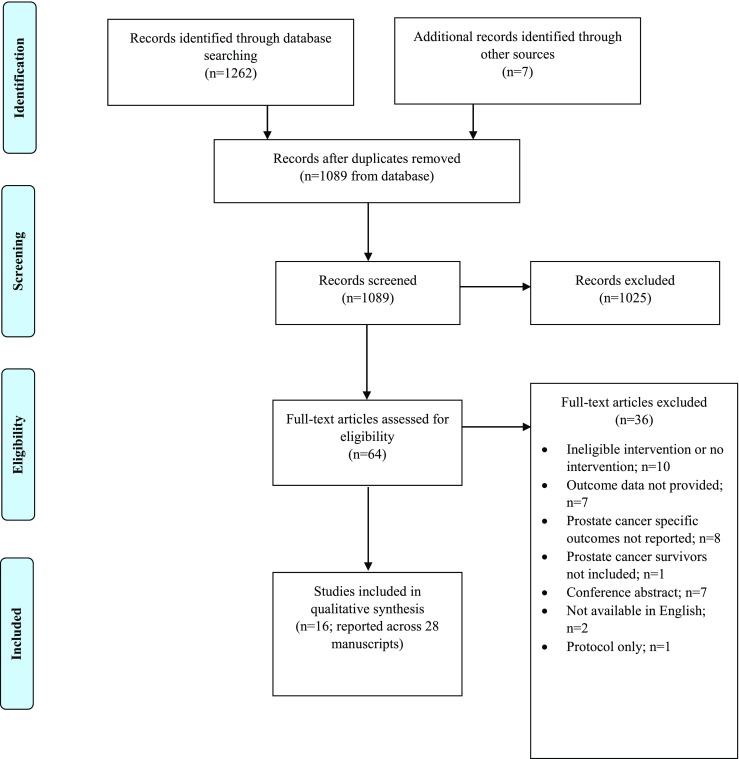
Prisma flow diagram

A flowchart of the study selection process is presented in Fig. 1. A total of 1269 publications were identified from all sources. After removal of duplicates, 1089 titles and abstracts were screened, of which 64 were included in the full-text review. Of those, 16 studies were identified as eligible and included in full data extraction for this review.

### Risk of Bias

Findings from the methodological review are presented in Table 1. Based on assessments from two reviewers, two of the studies [36, 37] received a global rating of “strong,” eight of the studies received a global rating of moderate [26, 35, 38–40, 43, 45, 48], and six received a global rating of “weak” [25, 41, 42, 44, 46, 47]. Studies with a weak rating tended to be small-sample single-arm studies designed to obtain preliminary insights into feasibility and acceptability.

**Table 1:** Risk of bias assessment summary

Author	Selection bias	Design	Confounders	Blinding	Withdrawals	Data collection methods	Global rating
Berry (2013) [36]	Moderate	Strong	Strong	Moderate	Strong	Strong	Strong
Berry (2017) [37]	Moderate	Strong	Strong	Moderate	Moderate	Strong	Strong
Cathala (2003) [38]	Weak	Weak	n/a	Moderate	Moderate	Weak	Weak
Davison (2014) [39]	Strong	Moderate	n/a	Moderate	Weak	Strong	Moderate
Diefenbach (2012) [40]	Moderate	Strong	Weak	Moderate	Moderate	Strong	Moderate
Fleisher (2015) [41]	Weak	Strong	n/a	Moderate	Moderate	Weak	Weak
Johnson (2016) [42]	Strong	Moderate	n/a	Moderate	Weak	Weak	Weak
Kazer (2011) [43]	Weak	Moderate	n/a	Moderate	Moderate	Strong	Moderate
Lange (2017) [44]	Weak	Moderate	n/a	Moderate	Weak	Weak	Weak
Osei (2013) [25]	Weak	Strong	Strong	Moderate	Weak	Strong	Weak
Ruland (2013) [26]	Weak	Strong	Strong	Moderate	Moderate	Strong	Moderate
Schover (2012) [45]	Weak	Strong	Moderate	Moderate	Strong	Strong	Moderate
Song (2015) [46]	Weak	Moderate	n/a	Moderate	Strong	Moderate	Moderate
Viers (2015) [47]	Weak	Strong	Weak	Moderate	Moderate	Moderate	Weak
Wootten (2015) [48]	Weak	Strong	Strong	Moderate	Moderate	Strong	Moderate
Yanez (2015) [35]	Weak	Strong	Strong	Moderate	Strong	Strong	Moderate

### Study characteristics

This review included 16 primary study papers [25, 26, 35–48] and a further 16 associated papers describing pilot studies, evaluations, and prior research that informed the primary papers [12, 15, 49–61]. Included studies were conducted in six different countries (11 in USA [25, 35–37, 40–43, 45–47], one each in Australia [48], Canada [39], France [38], Germany [44], and Norway [26]). Ten of the 16 studies were classified as RCTs [25, 26, 35–37, 40, 41, 45, 47, 48]; however, one did not report any efficacy outcomes [41]. The remainder comprised of pre/post-test cohorts [39, 42, 43, 46], and a single study using a two-group quasi-experimental design [44] and one single group evaluation [38]. Study duration ranged from less than 1 h to 1 year.

Fifteen studies exclusively targeted men with prostate cancer [25, 35–48]; one study included both breast and prostate cancer examined separately [26]. The total number of men with prostate cancer in all studies was 2446. Eleven studies included men with localized prostate cancer [36–43, 45, 46, 48], one with advanced/metastatic disease [35], and four were either unknown or did not report stage information [25, 26, 44, 47]. Treatment types reported were active surveillance [43], surgery [26, 38, 44–48], radiotherapy [26, 45, 46, 48], hormone therapy [26, 35], and not yet had treatment [36, 37, 39–42]; one study did not report treatment type [25]. Detailed information on the study characteristics can be found in Table 2.

**Table 2:** Description of study characteristics

Source (author and year)	Design	Location	Sample characteristics			Feasibility	
			Sample size; age	Disease stage	Treatment info	Recruitment rates	Retention rates
Cathala (2003) [38]	Single group evaluation, post-test only	France (% rural not reported)	*n* = 140; median: 63	Localized PC	100% radical prostatectomy	28% recruitment rate (140/508)*^np^	79% post-study measure (111/140)
Davison (2014) [39]	Single group, quasi-experimental design	Canada (57% rural)	*n* = 49; mean 60.5	Localized PC	No current treatment	92% (49/53) of patients referred agreed to participate*^np^	94% (46/49) follow-up
Johnson (2016) [42]	Single group, pre/post-test design	USA (% rural not reported)	*n* = 109; median: 62.5	Localized PC	No current treatment	87% (125/143) of men invited completed baseline surveys*^np^	87% (109/125) follow-up 26% (33/125) satisfaction survey
Kazer (2011) [43]	Single subject design	USA (% rural not reported)	*n* = 9; median: 72	Localized PC	100% active surveillance	45% (9/20) of sample target recruited^^nm^	67% (6/9) post-measures
Lange (2017) [44]	Two group, quasi-experimental design	Germany (26% village, 21% small town, 19% medium town)	Intervention *n* = 18; median: 60.5; control: *n* = 16; median: 62.8	Not reported	100% prostatectomy	11% (44/384) recruitment rate over 17 months (aimed for 120)^^nm^	31% (44/143) post-measures
Song (2015) [46]	Single group, pre/post-test design	USA (% rural not reported)	*n* = 26 couples; mean: 63 (PC)	Localized PC	41% surgery (type not specified) 59% radiotherapy	51% recruitment rate (26/51)*^np^	85% (22/26) post-intervention
Berry (2013) [36]	2-arm RCT	USA “Urban” (% rural not reported)	Intervention *n* = 266; median: 63 control: *n* = 228; median: 62	Localized PC	No current treatment	68% response rate (494/724)^^nm^	91% intervention vs. 87% control
Berry (2017) [37]	2-arm RCT	USA “Urban” (% rural not reported)	Intervention *n* = 198; 69.6% aged ≥ 60 years control *n* = 194; 66.7% aged ≥ 60 years	Localized PC	No current treatment	70.8% response rate (392/554)#^m^	76% (152/198) intervention vs. 79% (153/194) control
Diefenbach (2012) [40]	3-arm RCT	USA “Urban” (% rural not reported)	Interventions T-PIES: *n* = 32; mean: 60 NT-PIES: *n* = 21; mean: 63 control: *n* = 19; mean: 64	Localized PC	No current treatment	75% (91/121) eligible men agreed to participate*^np^	79% (72/91) post-measures
Fleisher (2015) [41]	2-arm RCT	USA (% rural not reported)	Project 1: (PC only) *n* = 439; mean: 65	Localized PC	No current treatment	61% (*n* = 439) of accrual target met^^nm^	77% (339/439) follow-up
Osei (2013) [25]	2-arm RCT	USA (% rural not reported)	Intervention: *n* = 20; control *n* = 20; overall mean: 67	Not reported	Not reported	5% response rate (51/1000) for expressions of interest*^np^	100% post-intervention
Ruland (2013) [26]	2-arm RCT	Norway (% rural not reported)	Intervention: *n* = 162 (96 breast and 66 prostate); mean: 57 control: *n* = 163 (93 breast and 70 prostate); mean: 56	10–12% metastatic disease*not PC specific	8% prostatectomy 26% radiotherapy 48% hormone therapy	Unknown response rate. 445 people expressed interest and 325 (73%) consented and randomized#^m^	Overall 77% intervention vs. 84% control group PC only 83% intervention vs. 84% control 81% PC survivors at 12 months
Schover (2012) [45]	3-arm RCT	USA “Urban” (% rural not reported)	Interventions FF: *n* = 60 couples; mean: 64 WEB1: *n* = 41 couples; mean: 64 WEB2: *n* = 71 couples; mean: 64 Waitlist control *n* = 48, then randomized to FF (*n* = 20) or WEB1 (*n* = 22)	Localized PC	74% radical prostatectomy 26% radiotherapy	Unknown response rate, no pre-specified sample size*^np^	72% FF vs. 87% WEB1 vs. 83% WEB2 post-intervention 93% FF vs. 83% WEB1 vs. 73% WEB2 follow-up 9% drop-out in WL group during 3 month waiting period.
Viers (2015) [47]	2-arm RCT	USA (% rural not reported)	Intervention VV: *n* = 34; mean: 62.5* OV (control): *n* = 36; mean: 61.5** those having completed post measures	Not reported	100% radical prostatectomy	31% (70/225) of group initially contacted randomized #^m^	79% (55/70) follow-up
Wooten (2015) [48]	3-arm RCT	Australia (15–18% inner regional)	Interventions MRA only: *n* = 47; MRA + Forum: n = 48; Forum only (control): *n* = 47; overall mean: 61	Localized PC	86% radical prostatectomy 13% EBRT 6% hormone therapy (in conjunction with RT)	95% (142/150) of recruitment target met ^^nm^	87% midweek follow-up 73% post-intervention 66% 3-month follow-up [60] 51% 6-month follow-up [60]
Yanez (2015) [35]	2-arm RCT	USA “Urban” (% rural not reported)	Intervention *n* = 37; control *n* = 37 Overall mean: 69	49% advanced 51% metastatic	100% on ADT	13% (74/240) of recruitment rate target met^^nm^	86% intervention vs. 86% control follow-up

*ADT* androgen deprivation therapy, *PC* prostate cancer, *RCT* randomized controlled trial, *FF* face-to-face, *PIES* prostate interactive education system, *T-PIES* tailored PIES, *NT-PIES* non-tailored-PIES, *UC* usual care, *V-CIS* virtual cancer information service, *WL* wait-list, *FF* face-to-face, *OV* office visits, *VV* video visits, *EBRT* external beam radiation therapy

*^np^Recruitment target not provided

^^nm^Recruitment target not met

#^m^Recruitment target met

### Intervention characteristics

Six of the evaluated studies were one-time interventions designed to improve knowledge and reduce decisional conflict prior to clinician visits [36, 37, 39–42]. Two interventions were designed to replace office visits; one 1-time “video visit” with the urologist [47] and one online medical record intervention, where patients could review their record and report symptoms for their doctor to review [38]. One 5-week intervention aimed to reduce uncertainty and increase self-care management among men during active surveillance [43]. Another 5-week intervention had men participate in one counseling session per week with the purpose of improving mental health [44]. An 8-week intervention for couples was designed to increase symptom management and communication skills [46]. A number of interventions aimed to improve one or more aspects of QoL and reduce distress; one 6-weeks [25], two 10-weeks [35, 48], one 12-weeks [45], one 1-year in length [26].

All interventions used some form of targeted or tailored education [25, 26, 35–48], nine had expert involvement in the form of feedback or counseling [26, 35, 38, 43–48], three had interactive exercises or homework to complete [45, 46, 48], three had self-tracking for symptoms which would be evaluated by a health care practitioner [26, 38, 47], seven had aspects of social support in the form of chat groups, communication skill building, or videos [25, 26, 38, 41, 43, 46, 47], seven taught stress reduction or coping techniques [35–37, 41, 43, 45, 46, 48]. The majority were website-based interventions with one study using video conference to deliver the study instead [47], one incorporating CD-ROM options [41], and one delivered specifically via tablet using video conference [35]. Very few studies specified following a theory or framework when developing their interventions. Two studies indicated self-regulation theory guided them [40, 41] and two others indicated they structured the intervention following Cognitive Behavior Therapy frameworks [35, 48].

### Feasibility and acceptability

Based on previous research among cancer patients [11, 35], six studies did not meet acceptable recruitment rates of 40% [25, 35, 38, 44, 45, 47], while three did not meet acceptable retention rates of 70% [43–45]. The average recruitment rate of 15 studies was 54% (ranging from 5 to 95%); one study did not report a response or recruitment rate [45]. The average overall retention rate was 78% (ranging from 31 to 100%). Of the 16 studies included, 9 reported a recruitment goal [26, 35–37, 41, 43, 44, 47, 48], of which 3 studies indicated meeting their goal [26, 37, 47]. Seven studies reported problems with recruitment [25, 36, 41, 43, 44, 46]. Potential reasons for recruitment issues were reported to be having a small number of men to sample from within a urological practice [43], too stringent eligibility criteria [25], wariness of technology [25, 44], burden of time commitment at stressful time [41], and call center recruitment issues [41]. In addition, one study indicated a large number of participants were lost after baseline measures due to long wait times getting the study started [44]. Few studies made any mention of implementation costs [25, 26, 38]; only one indicated the monetary cost of implementing the study [38]. Overall, the majority of studies required at least basic administrative time and maintenance on the part of either the researchers or the health care professional. Five studies reported intervention URLs [25, 26, 38, 44, 48] of which two [25, 44] were still active at time of data extraction. Detailed information on study feasibility can be found in Table 2.

Due to the variety of study designs apparent, intervention adherence was not assessed across all studies. Eight studies reported a percentage of participants that adhered in some way to the study parameters; the average “adherence rate” being 68% [35, 39, 41, 42, 45–48]. Outcomes of study adherence reported included participation in online sessions or modules [35, 43, 44, 47, 48], completing “homework” or extra modules [35, 45, 46], using a decision aid to completion [39], and sharing a decision aid summary page with a health care professional [39]. Usage data collected included time spent on a website, chat group or with a decision aid [35, 41, 44, 46, 59, 61], number of visits to a program or website [26, 38, 41, 43, 45, 46], and number of messages sent to health care provider or posted on a forum [26, 38, 48].

Most studies included one or more general measure of participant satisfaction [25, 35–39, 41, 43, 44, 46], with most participants reporting they were at least moderately satisfied with the program or intervention. Four studies reported high satisfaction with the quality of their cancer care rather than the intervention itself [40, 42, 46, 47]. One study did not report any satisfaction measures [26]. Four studies indicated that participants reported some kind of technical difficulty [38, 41, 47, 48], including incorrect data entry [40], absence of required software [12], general technical difficulties [41, 47, 61], and not knowing how to use the software [41]. No adverse events were reported in any study. Detailed information on the acceptability of the programs can be found in Table 3.

**Table 3:** Intervention overview and engagement and acceptability outcomes

Source (author and year)	Objectives and description of intervention	Engagement (usage)	Acceptability (satisfaction)	Conclusions
Cathala (2003) [38]	Objective: test and evaluate online medical file in lieu of face-to-face visit Participants could login to the EHR to view various hospital reports. They could view videos and read information regarding their operation and condition. They could complete sections that allowed them to track their PSA levels and their QoL over time. Any new entries would trigger an email to the chosen physician.	First 6 months tracked by the “connection report system” 95% regularly looked at the site 8 (mean) connections per patient (range 1–22): 2 dialogue zone messages 2 PSA entries 4 QoL surveys	98% satisfied with various site sections 94% satisfied with medical file 11% had problems accessing the site 14% reported technical problems	The online medical program approach was determined to be useful and acceptable for those patients requiring regular follow up. It overcame geographical barriers and allowed close contact between patients and health professionals, while also allowing physicians access to medical files.
Davison (2014) [39]	Objective: to prepare and identify treatment preferences and reduce decisional conflict during selection of PC treatment. Participants used an online decisional aid to produce a summary page intended to be presented to a clinician before treatment begins. The summary identified their personal preferences around decisional control, type and amount of information wanted, factors influencing their decision, and their preliminary treatment choice.	61% shared summary sheet with a health care worker involved in care: 35% with urologist 14% radiation oncologist 12% with family doctor 47% with nurse educator	Patients satisfied with amount of information (96%), type of information (93%), way delivered (89%), involvement in decision (91%), and treatment choice (96%) Participants that shared summary with their HCP were significantly more satisfied with the usefulness of the sheet.	The use of this support aid was found to be acceptable to patients, for use at home or in clinic. This can assist men with localized PC to identify the factors having an influence on their treatment decision and provides a means for these men to share these preferences and values with their physician at the time of treatment discussions. This simple tool could also easily be incorporated into clinical practice in order to guide treatment discussions provided by oncology nurses to the patient group.
Johnson (2016) [42]	Objective: to support patients with shared decision-making and reduce decisional conflict for men with newly diagnosed LPC prior to treatment decision. WiserCare was a web-based application that provides education, preference measurement and personalized decision analysis. A report was generated that was included in the patient file to be reviewed by clinicians before consultation.	76% of men invited voluntarily completed the decision support module 125 patients who clicked on the link completed the module. 109 (87%) completed the module and the follow-up survey	82% mostly satisfied or delighted with quality of care 97% mostly satisfied or delighted with explanations of treatment and procedures 97% mostly satisfied or delighted with helpfulness of information	Implementation of the WiserCare application was found to be feasible and improved several important components of decision quality for men deciding on treatment for newly diagnosed LPC. Compared to similar patients who did not participate in WiserCare, patients who completed this decision aid felt more included in and jointly responsible for their treatment decisions, and strongly agreed that treatment decisions were discussed in detail by their provider.
Kazer (2011) [43]	Objective: to support patients to improve knowledge and self-efficacy, and reduce uncertainty during AS for PC. Intervention delivered (a) general information about PC and AS; (b) cognitive reframing strategies; (c) self-care management strategies; and (d) tailored email-based interventions specific to the needs of each participant.	Average 20 website views per participant (range 2–40).	4.2 out of 5 for overall satisfaction of website and information	The study findings showed positive trends in acceptability of the trial. A larger clinical trial is planned to follow this pilot.
Lange (2017) [44]	Objective: to support mental health (e.g. reduce distress and improve QoL) in PC survivors. Participated in 5 group-based online chat sessions (60–90 min each, once per week over 5 weeks) led by a certified psychotherapist with experience in psycho-oncology. Each session had a theme proposed by the therapist but open interaction among participants was also encouraged.	100% of intervention group completed the evaluation session No report of participant engagement with chat groups The frequency and duration of sessions was “sufficient” (scores of 2.06 each). “Lack of interest” (31%) and “doubting that it could help me” (31%) were the most frequent reasons for non-participation	Overall positive evaluation of program (1 = strongly agree; 5 = strongly disagree) 2.44/5 – overall satisfaction with chat program 2.61/5 – therapist helpful 4.39/5 – had problems with chat program 3.95/5 – had computer problems during chat	The study findings indicate that web based chat groups may not be an effective way to decrease PC perceived distress despite apparent user acceptance of the intervention. Study highlighted the difficulty in recruitment and engagement of patients even in a major prostate cancer center.
Song (2015) [46]	Objective: to support patients’ knowledge of symptom management, communication skills, and improve QoL in LPC post-treatment patients. Couples were provided seven education modules to review (two were mandatory, and five were optional). Mandatory modules provided information about how couples can work as a team (e.g., communication) and survivorship issues. Optional modules focused on the management of PC-specific symptoms and general symptoms.	No report of participant engagement in mandatory modules. Average number of logins: 2.73 per patient Average total time spent on PERC intervention was 41.99 min Optional modules completed: 77% sexual dysfunction 77% fatigue 76% urinary dysfunction	Overall positive evaluation of program (1 = strongly disagree; 5 = strongly agree) 4.41/5 website easy to use 4.14/5 website was interesting and engaging 3.09/5 satisfied with quality of information	The study found the intervention (PERC) was a feasible and acceptable method of reducing side effects of PC treatment-related symptoms and improving QoL. Addition, participants rated PERC as easy to use and understand, and they found it to be engaging, high quality, and relevant. High usage rates were encouraging, particularly as PERC targeted older adults (with traditionally poor technology literacy). This method may be useful for overcoming geographical barriers and improving the convenience of information access for patients.
Berry (2013) [36]	Objective: reduce uncertainty and decisional conflict during selection of PC treatment. The intervention was a treatment decision aid tool with tailored information based on important identified personal factors, age, race and ethnicity, decisional control preferences, and symptoms. Participants spend time on the site, engaging with the education and assessments. Pre- and post-questionnaires are minimal. Baseline session involved surveys to determine personal preferences, factors, symptoms, decisional conflict, etc. Once complete, participants were randomized to one of two groups by an algorithm embedded in the software. P3P is composed of education and communication coaching. This takes the form of text, graphs, video clips, infographics, etc. Tailored to personally relevant factors assessed at baseline. For example, if a participant indicated sexual health a priority then this would be one of the first topics discussed. Post-measures 1-month later were completed via email or mail; whichever was preferred. DC was assessed again.	Time to complete average 46 min (range 16 to 69 min) [50]	(Higher score = more positive) [50] 4.1/5 overall satisfaction 4.8/5 easy to use 4.7/5 understandability 4.0/5 helpfulness of program 4.0/5 enjoy program 3.7/5 value of information Current study: 3.7/5 average usefulness	P3P is a useable and acceptable decision support system that can be deployed in a clinical setting [50] P3P did not result in higher preparation for decision making at 1 month. Satisfaction with decision was not associated with intervention use at 6 months [51].
Berry (2017) [37]	Objective: reduce uncertainty and decisional conflict during selection of PC treatment. Updated from Berry (2013) [36] to be more appropriate for lower literacy levels.	Pilot study [59] used eye tracking to determine “time to first fixation” and “total visit duration” in mean seconds on various aspects of the page (*n* = 12). Time to first fixation: M (SD) Text “understanding statistics” 8.9 (19.5) Text: YTL 24.1 (19.4) Infographic 41 (97.9) Total visit duration: M (SD) Text “understanding statistics” 39.7 (32.9) Text: YTL 53.8 (31.7) Infographic 21.7 (13.7)	(Higher score = more positive) [59] 4.8/5 ease of use 4.5/5 enjoyment 4.7/5 helpfulness 4.7/5 satisfaction 4.6/5 amount of time acceptable 4.9/5 understandable 4.5/5 usefulness of info	This aid to shared decision-making may be helpful to support men recently diagnosed with LPC.
Diefenbach (2012) [40]	Objective: to support patients to improve knowledge of treatments, side effects, self-efficacy, and QoL, and reduce uncertainty during AS for PC. T-PIES: participants were presented information according to their preferred monitoring style (high vs. low). Education was available via the library, and they could ask questions to a clinician and participate in a support group. They could also fill out a decision-making assessment if they wanted. NT-PIES: participants had access to the PIES system as above but written information was not tailored to the monitoring preferences of the user.	79% (72/91) completed post measures	Total sample (higher score = more positive) 4.15/5 useful information 4.15/5 satisfactory information 1.48/5 confusing information UC more likely to report: information was confusing (*p* = .05) too voluminous (*p* = .01), made them more anxious (less helpful *p* = .01; calmed nerves *p* = .04). Intervention groups only (no sig differences between intervention groups) (5 = strongly agree) 3.76/5 information clearly presented 3.46/5 includes everything I need 2.57/5 more information than I want 4.04/5 graphics clear 3.87/5 glossary helpful 3.80/5 library easy to understand 3.98/5 library useful	The implementation of PIES within a clinical practice was found to be feasible and acceptable to patients recently diagnosed with prostate cancer.
Fleisher (2015) [41]	Objective: to support patients to improve knowledge of treatments, side effects, and promote informed decision-making for newly-diagnosed PC patients. The intervention consisted of four modules: Library – text-based information on a variety of relevant topics (e.g., what is prostate cancer, treatment options, clinical trials). Patient stories – multi-media testimonials with actual patients were used and presented either with video or still photographs with voice-overs focusing on relevant issues, including treatment choices, side-effects, and emotional reactions. Doctor’s office – video-based and text-based information on cancer specific topics and FAQs as well as video demonstrations of how to communicate with providers using an evidence-based communication approach. Notebook – interactive values clarification tool where patients could list the pros and cons of each treatment approach and rank in terms of personal values.	96% in intervention group read the print materials 57% used the website and/or CD-ROM, of which: 54% used the website only 24% used CD-ROM only 22% used both 79% of website/CD-ROM users, reported using the V-CIS for an hour or more Participants who reported not using the V-CIS: 21% reported “no time” or “too busy” 25% said it was “not needed” 41% had technical problems Objective usage *n* = 32 (44%) 59% logged on more than once 96% accessed the library 50% accessed the patient story 59% accessed the doctors’ office 40.6% accessed the notebook Average overall time spent was 70.9 min (SD = 67.6)	Overall satisfaction (1 = strongly agree; 4 = strongly disagree) * significant differences across projects 1.69/5 increased knowledge^⁎^ 1.80/5 helped me talk to my doctor^⁎^ 2.01/5 helped with emotional concerns 2.98/5 made me feel more anxious^⁎^ 1.84/5 made me feel more confident^⁎^ 1.87/5 helped make treatment decisions^⁎^ 1.76/5 information helped me deal with cancer treatment^⁎^ 1.99/5 information helped me deal with side effects 2.25/5 helped me deal with emotional concerns of recurrence 2.18/5 helped me adjust to life after treatment 2.06/5 helped me understand my follow-up care	Feedback from VCIS users indicated that it significantly increased their self-reported knowledge, helped them to talk with their doctor, lower anxiety and increase confidence, and helped make treatment decisions. Cancer patients value both print and eHealth interventions. Barriers to adoption and usage remain, such as lack of perceived need and issues with technology.
Osei (2013) [25]	Objective: to support and improve patients’ knowledge (of treatments and side effects) and QoL in patients diagnosed with PC. Intervention utilized the online education and support group - Us TOO International Web site. Participants were asked to participate in the group at least three times per week for 6 weeks.	Study did not report actual usage. Participation was not defined therefore it is unclear whether men had to comment, post, or just read/view educational material to be considered participating.	Overall program satisfaction (4-point scale) Quality of services 75% good or excellent Received type of services wanted 89% generally/definitely Needs were met 21% all needs met, 37% most needs met 3.01/4 overall satisfaction Four men made statements about the need for other prostate cancer patients to get this information.	It is unknown as to the effectiveness of online vs. face-to-face groups, whether online support is contraindicated based on specific patient psychosocial characteristics, and the role that physicians play in effectiveness based on their recommendation.
Ruland (2013) [26]	Objective: to support and improve patients’ knowledge and QoL, and lower symptom distress in patients diagnosed with PC. Participants were given access to WebChoice online intervention which included: - assessment component - tailored symptom self- management support - information section - communication section (forum and Q&A with nurse) - personal diary Participants were told that they could use WebChoice as often they liked during their 1-year study participation and that use of the system was entirely voluntary.	Activity log tracked server details by ID for participants. 2 reports were generated; usage and user report. Usage – within specific period of time: logins, section visits, total visit duration, messages sent, and forum posts. User – patient use of the system: section visits, number of assessments, number of messages sent, posts, and diary notes. PCS (*n* = 47/66) logged on at least twice were categorized as “users” [57] Users used the site 2018 times (median = 10.0; range 2–593 logons). Number of visits to sections (median) Discussion forum: 1409 (4.0) Message to nurse: 624 (4.0) Assessments: 622 (5.0) Self-management support: 348 (5.0) Information section: 271 (3.0) Diary: 308 (2.0)	A Likert scale from 1 (not at all useful) to 9 (highly useful) was used to evaluate the usefulness of information given to patients. PC specific data was not provided. [57] How useful Self-management interventions 6.1 (1.9) General information 6.5 (1.7) Discussion forum 6.4 (1.7) Answer from nurse 7.6 (1.6) Of what quality Self-management interventions 6.5 (1.6) General information 6.8 (1.6) Discussion forum 6.7 (1.5) Answer from nurse 7.6 (1.6) How easy to understand Self-management interventions 7.3 (1.5) General information 7.2 (1.4) Discussion forum 7.6 (1.4) Answer from nurse 8.1 (1.3)	The study found WebChoice to be a promising tool to help cancer patients better manage their illness and reduce symptom distress.
Schover (2012) [45]	Objective: to support and improve patients’ knowledge of and manage symptoms of erectile dysfunction, and improve sexual satisfaction in PC survivors. The intervention consisted of sexual counseling. Couples were randomized adaptively to a 3-month WL, a 3-session face-to-face format (FF), or an Internet-based format (WEB1). A second Internet-based group (WEB2) was added to examine the relation between web site use and outcomes. 3 homework reports required in each condition.	52% of men and 44% of partners completed > 75% of the web site Participants asked to engage in the site or attend 3 FF sessions over 12 weeks. Unknown homework report completion rate.	No measures of satisfaction were included.	The study found the Internet-based sexual counseling program for couples to be as effective as a brief traditional sex therapy format in producing persistent improvements in sexual outcomes after prostate cancer. The time required for therapists to respond to emails was significantly shorter than time required to conduct traditional therapy sessions, suggesting Internet-based interventions may be more time- and cost-effective as well as accessible to patients.
Viers (2015) [47]	Objective: to replace traditional patient visits and improve cost, efficiency and patient satisfaction in PC patients post-prostatectomy. VV from home or work with urologist. A mail-in PSA test was completed locally before the appointment. Patients were evaluated by a resident or midlevel provider and a staff urologist. If a physical examination was indicated, a follow-up clinic appointment was provided.	82% of those randomized to video appointment completed it.	No significant difference in patient satisfaction between the intervention (remote visits) and control (office visits) groups. “I was pleased with the quality of the medical encounter” 83%/91% strongly agree intervention/control (*p* = .41) “I believe that the medical encounter was conducted in a confidential manner” 88%/100% strongly agree intervention/control (*p* = .09) “I was overall satisfied with appointment today” 88%/91% strongly agree intervention/control (*p* = .70)	The majority (96%) of patients undergoing VV would participate in this type of encounter again. For established patients, this model could be applied across multiple urologic indications and clinical scenarios. VV timings improved across all measures throughout the course of the trial, with only two technical failures early in the study. Funding and credentialing limited the size of the study population. ~70% of physicians reported that credentialing is a significant setback to the implementation of telemedicine.
Wooten (2015) [48]	Objective: to support patients to improve their knowledge and reduce psychological distress in LPC patients undergoing treatment. Self-directed CBT-based intervention provided psycho-education, a series of interactive exercises and regular feedback. Intervention was a 6-module online program for participants to work through at their own pace over 10 weeks. The six modules focused on: 1. The emotional impact of prostate cancer 2. Cognitive strategies and effective communication 3. Coping with the physical challenges relating to prostate cancer 4. Sexuality and masculinity 5. Sexuality and intimacy 6. Planning for the future	59% (mean) content completed MRA only group = 60% MRA + forum group = 57% Completion rates dropped as participants moved through modules. On average participants completed: 87% module 1, 72% module 2, 60% module 3, 56% module 4, 41% module 5, 36% of module 6 Forum participation was higher for Forum alone group (avg. 2–3 posts per user) than MRA + forum (avg. 1–2 post per user) 69% of participants reported spending < 30 min per week on the forum.	Overall satisfaction *n* = 26 [61] 48% satisfied with intervention 78% would recommend Forum satisfaction 41% satisfied with forum 66% said easy to use 38% said other men’s posts were helpful 31% said moderator posts were useful As a result of these findings and qualitative feedback from users and technical consultants, changes were implemented in the larger included study. However, satisfaction results have yet to be published.	The intervention was received positively by participants in the pilot study. Feedback indicated good acceptability of the intervention. Some technical and participant engagement issues were identified and changes were implemented as a result of the pilot testing. The included study highlights the potential to deliver support for men with PC.
Yanez (2015) [35]	Objective: to support patients to ease their symptom burden and improve QoL in advanced PC patients. Intervention delivered via a web-based platform on a tablet by a group facilitator with video conferencing software. Intervention aims included developing stress awareness, learning stress reduction skills, changing negative stressor appraisals, developing coping skills, building interpersonal skills, and building or enhancing social networks. The website contained review materials of the principles of cognitive behavioral stress management (discussed during the weekly group meetings), as well as audio recording of relaxation strategies (e.g., guided imagery) that participants were encouraged to review and practice on a weekly basis.	CBSM-intervention group attended 6.59/10 sessions (SD = 3.35) HP-control group completed 8.22/10 (2.75) sessions CBSM-intervention group completed 4.84 (3.35) weekly assessments HP-control group completed 7.05 (3.14) weekly assessments During the first 30 min of each group session, CBSM participants practiced a new stress reduction/relaxation technique. During the last 60 min, the focus was on stress management.	No between group difference in exit survey scores (4-items). 4-point scale (higher numbers = more positive) Questions: mean (SD) In general, how much did you like the information presented in the weekly online reviews? CBSM: 3.65 (0.49) HP: 3.40 (0.76) In general, how much did you like the information presented in the online expert videos? CBSM: 3.66 (0.50) HP: 3.46 (0.52) In general, how much did you like the weekly online groups? CBSM: 3.40 (0.83) HP: 3.68 (0.69) In general, how much did you like the online relaxation exercises? CBSM: 3.81 (0.40) HP: N/A	Findings generally support the feasibility, acceptability and preliminary efficacy of this CBSM psychosocial intervention for men with advanced prostate cancer. Participants in HP condition were more likely to attend study sessions than participants in the CBSM condition.

*EHR* electronic health record, *PSA* prostate specific antigen, *PC* prostate cancer, *HCP* health care provider, *LPC* localized prostate cancer, *AS* active surveillance, *PERC* Prostate Cancer Education and Resources for Couples; P3P: Personal Patient Profile – Prostate, *DC* decisional conflict, *YTL* years to live, *PIES* prostate interactive education system, *T-PIES* tailored PIES, *NT-PIES* non-tailored-PIES, *UC* usual care, *V-CIS* virtual cancer information service, *WL* wait-list, *FF* face-to-face, *OV* office visits, *VV* video visits, *CBT* cognitive behavioral therapy, M*RA* My Road Ahead, *CBSM* Cognitive Behavioral Stress Management, *HP* health promotion, *QoL* Quality of Life

### Efficacy

Efficacy outcomes are reported for RCTs only. Outcomes assessed included decisional conflict [36, 37, 40], QoL [25, 26, 35, 48], distress [26, 35, 45], sexual function and satisfaction [45], relationship satisfaction [45], health care provider visit efficiency [47], psychological distress [48], depressive symptoms [26, 35], social cognitive outcomes [26], cancer-related symptoms [25], and treatment preferences [40]. Of the nine RCTs reporting efficacy outcomes, three reported significant improvements in the primary outcome relative to the control [25, 40, 48]. In addition, two reported a significant intervention effect on a subscale of the primary outcome [26, 36], and one reported significant intervention effects on secondary outcomes [37]. One study reported improvements in the primary outcome among all intervention groups, resulting in no significant difference between groups [45]. Of the remaining studies, three reported no significant intervention effects compared to the control group (which included face-to-face visits in most cases [35, 47]) and one did not report efficacy data [41]. Detailed information on the efficaciousness of the interventions can be found in Table 4.

**Table 4:** Summary of RCT results reporting study efficacy outcomes

Source	Design	Outcomes measured	Efficacy results	Conclusions
Berry (2013) [36]	2-arm RCT (*n* = 494) Decision aid tool vs attention control. Attention control given a list of reputable websites to review in the same time period as the intervention group.	Primary: decisional conflict subscales including: uncertainty, informed, values clarity, and support. Secondary: PC with decision at 6 months completed effective decision and total score.	Primary • Significantly less uncertainty (*p* = .04) and lack of values clarity (*p* = .002) in intervention group. • Subscales effect sizes: uncertainty: − 3.61 (− 7.01, − 0.22); lack of values clarity: − 3.57 (− 5.85, − 1.30) Secondary • No difference in total DC score between groups (*p* = .07). • Estimation of group study effect (coefficient (95%CI)): Total score − 1.75 (− 3.61,0.11); *p* = .07	Findings support efficacy of the P3P intervention for addressing uncertainty and facilitating selection of a treatment. P3P did not result in higher preparation for decision making at 1 month. Satisfaction with Decision was not associated with intervention use at 6 months [51].
Berry (2017) [37]	2-arm RCT (*n* = 392) Decision aid tool vs. attention control. Attention control given a list of reputable websites to review in the same time period as the intervention group. Decision aid updated from Berry (2013) to be more appropriate for lower literacy levels.	Primary: total score on modified low literacy decisional conflict scale (reported at baseline and 1 month)	Multivariate model: (LS mean (95%CI); *p*) • P3P vs. control: − 5.0 (− 9.40, − 0.59); *p* = .003 • Lower income: 8.69 (4.43, 12.96); *p* < .0001 • Having made no decision at 1 month: 20.11 (16.10, 24.13); *p* < .0001 • Lower D’Amico risk: 4.29 (0.80, 7.78); *p* = .02 Had ≥ 2 consults: 6.04 (1.83, 10.26); *p* = .0005 • EUH site: 11.02 (3.49, 18.56); *p* = .02• Interactions: Marginal significance for group and marital status (*p* = .06) with single men in P3P having lower DC. Marginal significance for number of consults by group (*p* = .07) with those in control group having < 2 consults having higher DC scores.	P3P demonstrated a beneficial effect for men with LPC in a multi-institutional sample as they engaged in decision-making for the management of the cancer. Other variables impacted conflict and modified P3P’s effect, notably risk level and men’s resources.
Diefenbach (2012) [40]	3-arm RCT (*n* = 72) T-PIES vs. NT-PIES vs. UC. T-PIES: information given according to preferred style of monitoring, and opportunity to ask clinicians, and a support group. NT-PIES: as above but written information not tailored to monitoring preferences. UC: attention control given NCI brochures to read in the same period as the intervention groups.	Primary: treatment decisional measures Secondary: psychological distress, treatment preferences	No difference between T-PIES vs. NT-PIES therefore PIES groups were combined for these comparisons vs control. PIES groups were significantly more confident in their treatment decision vs control. (Mean (SD): higher score better) UC: 3.22 (1.32) vs. PIES: 3.85 (1.022); *p* = .02 PIES groups less likely to report needing more information vs control. (Mean (SD): lower score better): UC: 3.44 (1.54) vs. PIES: 2.52 (1.49); *p* = .02 3 groups analyzed: UC vs. T-PIES vs. NT-PIES Helpful in decision-making (higher is better) UC: 1.79 (0.92) vs. T-PIES: 4.29 (0.64) vs. NT-PIES: 4.10 (1.07); *p* = .01 Calmed nerves about decision (higher is better) UC: 2.68 (1.06) vs. T-PIES: 3.12 (0.83) vs. NT-PIES: 3.46 (0.89); *p* = .04 Made more anxious about decision (lower is better) UC: 3.62 (1.05) vs. T-PIES: 2.45 (1.09) vs. NT-PIES: 2.40 (1.27); *p* = .03	PIES improved key decision-making process variables (e.g., knowledge of treatments and side effects), as well as increasing confidence in and reducing the emotional impact of a treatment decision making. No additional benefit to tailoring information to delivery style in the two intervention groups.
Osei (2013) [25]	2-arm RCT (*n* = 40) Online education and support group - Us TOO International Web site. Control group was given PC resource kits. Participants were asked to participate in the group at least three times per week for 6 weeks.	Primary: QoL (general and cancer specific, life satisfaction, relationship satisfaction) Secondary: prostate cancer-specific symptoms	MANOVAs mean differences across time No statistically significant effects of age and/or group but there was a significant (*p* = .036) time*group interaction across all ten measures included in a global QoL measure uniquely constructed for this study. Trend was for a drop in QoL at midpoint measure (6 weeks) with a return to initial levels at post measure. Variables affected included: perceived physical health (*p* < 0.001) urinary irritation and obstruction health (*p* < 0.019) sexual health (*p* < 0.001) hormonal health (*p* < 0.001) life satisfaction (*p* < 0.001) spouse negative characteristics (*p* < 0.030)	The results suggest that online support groups can have a positive effect on perceived QoL of men.
Ruland (2013) [26]	2-arm RCT (*n* = 325 overall; 136 PC) Intervention group (WebChoice users) vs. control (URLs of reputable websites)	Primary: symptom distress Secondary: depression, self-efficacy, health-related QoL, social support	Note: efficacy outcomes were not reported separately for breast vs. PC survivors. Reporting when applicable: slope estimate (95%CI); *t*; *p* • No between group difference in overall MSAS-SF score (*p* = .19) • No between group difference in psychological symptoms scale (*p* = .11) or physical symptoms scale (*p* = .09) • Significant between group difference in GDI: − 0.059; (− 0.101, − 0.004); *t* = 4.42; *p* = .037 • Within-group improvements in depression for intervention group: − 0.41; (− 0.71, − 0.11); *t* = − 2.71; *p* = .007 • Within group decrease in self-efficacy for control group: − 3.77; (− 6.38, − 1.15); *t* = − 2.82; *p* = .005) • Within group decrease in health-related QoL for control group: − 0.01; (− 0.01, − 0.00); *t* = − 2.77; *p* = .006	That WebChoice is a promising tool to help cancer patients better manage their illness and reduce symptom distress, is partially supported by the data. The secondary outcome measures did not show significant differences *between* study groups with respect to depression, self-efficacy, health-related QoL, and social support, however the benefits of WebChoice were still quite respectable. High use of symptom assessments, advice, and the discussion forum was associated with high levels of symptom distress [52].
Schover (2012) [45]	3-arm RCT (*n* = 182) 3-month waitlist (WL) vs. 3-session face-to-face format (FF) vs. 3 session internet-based format (WEB1). A second internet-based group (WEB2) was added.	Primary: erectile function for PC; female sexual function for partners. Secondary: emotional distress, relationship satisfaction	No significant changes in outcome measures during WL period Significant gains in IIEF for all men between baseline and 6 months (*p* < .0006) and 1-year (*p* < .0046) follow-up for erectile function. IIEF scores: *p* value, Cohen *d*: • FF across time *p* < .0001, *d* = 0.35 • WEB1 across time *p* = .004, *d* = 0.35 • WEB2 across time *p* = .0096, *d* = 0.27 No difference between groups (FF vs. WEB1 vs. WEB2) at follow-up for erectile function.	The internet-based sexual counseling program was found to be as effective as traditional face-to-face counseling for improving sexual outcomes in prostate cancer survivors.
Viers (2015) [47]	2-arm RCT (*n* = 70) Remote video visits (VV) from home or work with urologist vs. traditional office visits (OV).	Primary: visit efficiency (measured by time) Secondary: patient/provider satisfaction, cost of visits	No difference between groups in (VV mean vs. OV mean; (95% CI); *p*): • Total time in minutes devoted to patient care: 17.9 vs. 17.8; (− 5.9, 5.6); *p* = .97 • Total patient face time in minutes: 14.5 vs. 14.3; (− 5.4, 5.2); *p* = .96 • Patient–staff face time in minutes: 12.1 vs. 11.8; (− 4.2, 3.5); *p* = .85 • Patient waiting time in minutes: 18.4 vs 13.0, (− 13.7, 3.0); *p* = .20 Linear regression analysis of timing data revealed a downward trend in timing parameters for the VV arm, however, not statistically significant (*p* = 0.07) VV group had significantly lower estimated costs including (all *p* < 0.0001): • distance traveled (median 0 vs. 95 miles) • travel time (0 vs. 95 min) • work missed (0 vs. 1 day) • money spent ($0 vs. $48)	VVs had equivalent timing efficiency, similar patient satisfaction, and significantly reduced costs when compared to OVs. Specifically, VVs were associated with reductions in distance traveled, travel time, missed work, and money spent. A learning curve for the use of VV is present, but further investigations are needed.
Wooten (2015) [48]	3-arm RCT (*n* = 142) MRA only vs. MRA+ forum vs. forum only	Primary: psychological distress (DASS-21) Secondary: prostate cancer-related QoL, confidence	Pairwise comparisons • Psychological distress: MRA + forum (↓) vs. forum only (↑): − 8.8 (− 16.7, − 0.9); *p* = 0.02 • Informed decision: MRA only (↑) vs. MRA + forum (↓):15.3 (0.8, 29.8); *p* = 0.03 • Regret: MRA + forum (↓) vs. Forum only (↑): − 8.1 (− 16.1, − 0.1); *p* = 0.04 • Outlook: MRA only (↑) vs. Forum groups (↓): 17.2 (2.9, 31.4); *p* = 0.01	A statistically and clinically significant improvement in psychological distress was seen for participants who had access to both the online intervention and moderated forum, while no significant change in psychological distress was seen for the other two intervention conditions.
Yanez (2015) [35]	2-arm RCT (*n* = 74) CBSM (cognitive-behavioral stress management) intervention vs. health promotion attention control	Acceptability (main outcome detailed in Table 3) Secondary: cancer-related distress (IES-R), depressive symptoms, health-related QoL	CBSM mean vs. HP mean; *p* Completers Cancer-related distress: 8.39 vs. 10.20; *p* = .48 Depression: 43.37 vs. 47.29; *p* = .03 FACT total: 88.32 vs. 84.03; *p* = .17 Intention-to-treat Cancer-related distress: 8.46 vs. 9.86; *p* = .56 Depression: 44.04 vs. 47.13; *p* = .06 FACT total: 87.95 vs. 85.39; *p* = .39	Participants in HP condition were more likely to attend study sessions than participants in the CBSM condition. Although the sample size was underpowered effect sizes suggest the CBSM may have contributed to reduce depression more and reduce QoL more than control.

*PSA* prostate specific antigen, *PC* prostate cancer, *HCP* health care provider, *LPC* localized prostate cancer, *AS* active surveillance, *PERC* Prostate Cancer Education and Resources for Couples, *P3P* Personal Patient Profile – Prostate, *DC* decisional conflict, *EUH* Emory University Hospital, *YTL* years to live, *PIES* prostate interactive education system, *T-PIES* tailored PIES, *NT-PIES* non-tailored-PIES, *QoL* Quality of Life, *NCI* National Cancer Institute, *MSAS-SF* Memorial Symptom Assessment Scale-Short Form, *GDI* global distress index, *UC* usual care, *V-CIS* virtual cancer information service, *WL* wait-list, *FF* face-to-face, *IIEF* International Index of Erectile Function, *FSFI* Female sexual function index, *OV* office visits, *VV* video visits, *CBT* cognitive behavioral therapy, *MRA* My Road Ahead, *CBSM* Cognitive Behavioral Stress Management, *HP* health promotion, *IES-R* Impact of Event Scale–Revised, *ANOVA* analysis of variance, *ANCOVA* analysis of covariance, *MANOVA* multiple ANOVA

#### Decision making

Participants who received decision aids were more satisfied with their care and treatment decisions than those receiving standard care [36, 37, 39–42]. Three studies reported completing decision aids increases in self-reported knowledge, more confidence in their decision, and decreased uncertainty and decisional conflict compared to usual care [36, 37, 40, 41]. Participants that received a decision aid were less likely to be anxious about their decisions [40, 41]. Two studies reported that decision aids reduced distress [40, 41].

#### Quality of life

Participants that participated in an online support chat group reported improvements in QoL over time after treatment-related declines [25]. Participants that accessed a supportive online intervention reported improvements in QoL measures but not significantly different from the control group [26]. Access to cognitive behavioral therapy-based intervention only had an improvement in outlook compared to the forum groups. Those in the therapy group with forum access had reductions in regret compared to the forum only group indicating positive impact on PC-related QoL [48].

#### Sexual health

Participants that received internet-delivered sexual counseling had similar improvements in erectile function compared to face-to-face counseling [45] with no significant difference between the groups.

#### Mental health

Participants receiving access to an online supportive care intervention had improvements in depression measures [26]. Those that had access to a cognitive behavior therapy-based intervention had significantly better improvements in psychological distress than those with access to a chat forum only [48]. Another study that delivered cognitive behavioral stress management found no difference in cancer-related distress between groups but a trend toward reduced depressive symptoms in intention-to-treat analyses [35].

#### Visit efficiency

Studies that examined using online follow-ups or visits rather than face-to-face were also well received by participants [38, 47]. One study examined the efficiency and cost-effectiveness of video visits with an urologist rather than office visits and found no significant difference in any timing measure between the groups and estimated significantly less cost associated with the video group [47]. One study reported the amount of time for therapists to respond to email queries was less than time taken to conduct traditional therapy sessions [45].

## Discussion

The aim of this systematic review was to examine the feasibility, acceptability, and efficacy of online supportive care programs for men with prostate cancer. Overall, the results showed that using online delivery can be feasible and acceptable to men with prostate cancer; however, the field is still in its infancy. We found 16 studies that met our criteria among which 10 were randomized controlled trials. The results showed trends toward the programs being efficacious; however, among these trials, few were large enough to make meaningful conclusions on the efficacy of online supportive care programs, and selection bias was a consistent issue.

Though the average recruitment rate was 54%, only three of seven studies reporting recruitment goals met their goals. Recruitment is often a challenge in research studies among cancer survivors, particularly those targeting men [12, 62]. Collaborating with other centers to conduct multicenter trials may help to improve this to some extent. As well as increasing the recruitment pool, this may also help to recruit more representative samples, by ensuring participants are recruited from different geographical locations [63]. Another option would be to use multimodal recruitment strategies, such as social media ads combined with clinic-based recruitment as this has been shown to have similar advantages [26, 48]. As the majority of studies reviewed in this paper suffered from selection bias it is likely that the included sample is not entirely representative of the intended target group. Acceptability and efficacy findings should be interpreted with this in mind. In this sense, these results indicate that using online delivery for supportive care programs is feasible and acceptable, at least in some sub-groups of men with prostate cancer.

Despite the growing body of literature investigating online methods of providing patient support, we found only 16 studies met our inclusion criteria. There was little research among men with prostate cancer despite there being evidence of interest in supportive care programs among this population [16, 64, 65]. Furthermore, a large portion of the studies were testing decision aids for men who had localized prostate cancer and were yet to have any treatment. As one study points out, guidelines from the American Urological Association indicate that shared decision making is an important component of treatment counseling for men with localized disease [37]. These aids were seen as beneficial for men in increasing their knowledge about prostate cancer treatments and what treatment may be right for them, thereby reducing decisional conflict and regret. Only one study focused on men with advanced prostate cancer [35]. This restricts the generalizability of the conclusions to men with less severe forms of cancer.

While assisting men with prostate cancer to make informed treatment decisions is an important area of research, there are aspects of cancer care that have yet to be comprehensively addressed in this population. One area that warrants further attention in particular is the delivery of behavior change support. The studies included in this review focused mainly on psychological aspects of well-being, such as reducing distress, improving stress management and communication skills, and relationship satisfaction. However, activity levels, diet, and sleep behavior also impact QoL (both overall and disease specific) across the cancer continuum [66–70]. Structured exercise and physical activity in general have been shown to counteract prostate cancer related treatment toxicities, reduce disease progression in those with early stage disease, as well as improve psychosocial outcomes, and increase men’s sense of empowerment and control [69–72]. Additionally, online programs targeting physical activity and diet have been shown to be efficacious in other groups of cancer survivors [73]. It may be the case that interventions which encourage behavior change, such as physical activity, diet, and/or sleep, may be more effective and appealing to men than traditional psychological support, given that the outcomes (particularly those associated with exercise) often align with traditional masculine values [70]. However, additional high-quality research, particularly in prostate cancer, which assesses these outcomes objectively and longitudinally are required to not only establish their efficacy in targeting behavior change, but also supportive care needs [24, 73].

Summarizing results was difficult among the included studies as even those that focused on similar outcomes had measured or reported them differently. In addition, reporting of methods was lacking in many of the studies. As noted in Table 1 that summarizes the methodological review, only one study received a strong rating, five ranked weak and the remaining ten were ranked moderate. In order to grow this area of research, methods need be rigorously documented, as previous reviews have suggested [12, 14, 15, 62]. Lessons learned from previous research can greatly impact the body of literature by ensuring future studies build on what has been done previously.

While this study has been the first to summarize online supportive care interventions for men with prostate cancer, there are some study limitations that need to be mentioned. We understand that including only studies published in English reduced potential access to the total number of globally published studies. Furthermore, this study contained a high proportion of one-time clinical treatment decision support tools, and most studies had small samples or were pilot trials. This reflects the lack of variety of studies available, likely due to the infancy of this field and known issues with recruitment. Strengths of this study include a-priori protocol registration, the use of a standardized data-extraction form, the depth and range of data extracted, and synthesized and corroboration and consensus between a number of researchers during the data extraction and bias tool implementation. This allowed balanced assessments in which studies were fairly examined during the extraction and quality assessment stages of the review.

Aligned with previous research, we see a need for rigorous study development and reporting [11]. Methodological quality was generally weak mainly due to underreporting of methods. In order to build on or replicate results, clear description of the intervention components is necessary. Additionally, future research should ensure usage and adherence of individual intervention components are well reported. The majority of studies in this review included patients with localized disease. To address these research gaps, more focus should be on men with advanced disease and their specific supportive care needs.

Online supportive care may be particularly useful for clinicians as both decision aids and as a tool for patient follow-up. Many decision aids were able to be completed while waiting for a clinician or at home before an appointment. This allows men time to thoroughly examine the information and by sharing a report with their clinician, be more involved in the decision-making. For clinicians, this means less time devoted to treatment explanation in appointments and an increased feeling of shared decision making. Additionally, more than one study indicated that using online methods of follow-up, when possible, was just as efficient as office visits [25, 38, 47]. Clinicians spent the same, or fewer, minutes interacting with patients with no perceived reduction in quality of care. These methods may be more cost-effective for both clinicians and patients.

This review provides preliminary evidence in modest support of online supportive care programs for men with prostate cancer. Our conclusions are limited by the small number and weak methodological quality of studies found. A consistent call for well-documented, rigorously conducted studies has been noted in previous reviews and is echoed here.
